# Rising House Prices, Falling Fertility? How Rising House Prices Widen Fertility Differences between Tenure Groups

**DOI:** 10.1007/s10680-025-09754-6

**Published:** 2025-11-14

**Authors:** Daniël van Wijk, Peteke Feijten

**Affiliations:** 1 Netherlands Interdisciplinary Demographic Institute, KNAW/University of Groningen, The Hague, The Netherlands; 2 Statistics Netherlands, The Hague, The Netherlands

**Keywords:** Fertility, House prices, Homeownership, The Netherlands, Inequality

## Abstract

House prices rose rapidly in rich societies over the past decade, inhibiting young adults’ access to affordable, family-friendly housing. Over the same period, fertility has declined. Some recent studies have examined the connection between these trends, but the individual-level mechanisms that link house prices to fertility remain underexplored. We address this research gap by using register data on the full population of the Netherlands between 2012 and 2023, a period during which house prices increased dramatically. We link variation in changes in house prices across NUTS-3 regions to yearly conception risks and examine the mediating and moderating role of individual-level homeownership. Results show that increasing house prices are associated with lower fertility, which can partly be explained by the lower propensity of young adults to be homeowners and partly by decreased fertility among renters in more expensive housing markets. In contrast, increasing house prices increase the fertility of homeowners. This positive home equity effect is found only among those who entered into homeownership more than three years ago. These results indicate that rising house prices have likely contributed to the fertility decline observed after 2010 among younger cohorts and may amplify fertility differences between housing market insiders and outsiders.

## Introduction

“[T]he lack of attention to housing in population research stands in unjustified contrast to the routine attention paid to education and socioeconomic status” (Mulder, 2006:410).

This statement was written by Clara Mulder nearly two decades ago. Since then, scholars in demography (e.g., Kulu & Steele, 2013; Van Wijk, 2024), housing studies (e.g., Flynn, 2017), and—particularly—economics (e.g., Atalay et al., 2021; Dettling & Kearney, 2014; Liu et al., 2023) have devoted more attention to studying the links between housing and population patterns and developments, including fertility outcomes. However, the role of housing remains understudied in fertility research compared to other factors of interest. For example, a scan of the volumes of six high-ranking journals in population studies^1^ published between 2021 and 2024 reveals that out of a total of 278 studies on fertility, 58 focused on the role of educational enrollment and attainment, 62 focused on economic indicators such as employment and income, and only four (Florida et al., 2021; Lim et al., 2021; Tocchioni et al., 2021; Van Wijk, 2024) focused on housing. This relative shortage of studies focused on housing in fertility research is surprising given recent trends in rich societies, which should place the links between housing and fertility at the center of demographic attention. Fertility has declined since 2010 in many rich societies despite the fact that economies recovered from the 2007–2008 financial crisis, and in many countries the total fertility rate (TFR) reached record-low levels in recent years (Human Fertility Database, 2023). During the same period, house prices increased dramatically in nearly all OECD countries, as evidenced by rising real house prices and house price-to-income ratios (OECD, 2025). Combining these trends, it has often been suggested that the inability of many young adults to find affordable, family-friendly housing is making them postpone having children or even put off childbearing altogether (Galster & Lee, 2021; Kalleberg, 2018; Skirbekk, 2022). However, the number of recent studies that has empirically tested this expectation is small and has produced mixed results, with some reporting a negative relationship between house prices and fertility (Florida et al., 2021; Japaridze & Sayour, 2024; Liu et al., 2023; Van Wijk, 2024) and others a positive one (Atalay et al., 2021; Clark & Ferrer, 2019). Moreover, much remains unknown about the individual-level mechanisms that link house prices to fertility.

In the present study, we address this research gap by studying how (changes in) regional house prices affect individual-level fertility. We disentangle the mechanisms behind the house price effects on fertility by examining the mediating and moderating roles of homeownership. First, we expect that rising house prices prevent young adults from buying a home. Owner-occupied homes frequently come with other characteristics that are deemed appropriate for raising children (e.g., a spare room for each child, a garden), and homeownership signals adult status and financial stability. Therefore, obstacles to buying a home may inhibit childbearing decisions, in line with recent findings that show that fertility is lower among renters than among homeowners (Chudnovskaya, 2019; Flynn, 2017; Japaridze & Sayour, 2024; Lim, 2021; Tocchioni et al., 2021). As a result, rising house prices may decrease fertility by obstructing the transition to homeownership. However, we could not find any previous work that empirically examined this mediating role of homeownership.

Second, rising house prices augment inequalities between homeowners and renters, both in terms of the accessibility of housing and the opportunities to accumulate wealth (Boelhouwer, 2020). As a result, house prices may affect the fertility of renters and homeowners in different ways. For renters, rising house prices imply worse accessibility of family-friendly housing and higher housing costs, likely reducing fertility. For homeowners, a similar negative effect as a result of increasing costs may be relevant, but increasing house prices may also increase fertility by raising the level of wealth. In line with this expectation, a small literature in economics has shown that increases in housing wealth generally increase the fertility of homeowners, whereas the effects among renters—who do not profit from increased housing wealth—are absent or negative (Atalay et al., 2021; Clark & Ferrer, 2019; Dettling & Kearney, 2014; Lovenheim & Mumford, 2013; but see Liu et al., 2023). Although these studies provide interesting insights into the role of home equity in fertility decisions, they have mostly been concerned with the identification of a causal effect of housing wealth for a selective group of homeowners rather than uncovering house price effects in the general population. We build on these studies by examining how individual-level homeownership moderates the relationship between regional house prices and fertility. Furthermore, we add a distinction between recent and longer-term homeowners, as the extent of increases in both housing wealth and housing costs among homeowners likely depends on whether individuals transitioned to homeownership before or after the recent house price increases.

Our study is set in the Netherlands, a country that has been described as a career homeownership regime (Mulder & Billari, 2010) where the transition to homeownership often occurs around the time of family formation (Feijten & Mulder, 2002; Mulder & Wagner, 2001). This may indicate the presence of a norm that favors childrearing in owner-occupied housing. However, sharp increases in house prices have put pressure on this model. House price-to-income ratios in the Netherlands have increased by more than 30% since 2015, much more than the average increase in the euro area (OECD, 2025). Yet the magnitude of house price increases differs markedly across the country (Van Wijk, 2024), and we exploit these geographical differences in house price increases to investigate the impact of house prices on fertility. Taken together, these factors make the Netherlands a highly interesting case to study the effects of house prices on fertility. At the same time, the difficulties in accessing affordable, family-friendly housing faced by the current cohort of young adults are by no means unique to the Netherlands, and the present study has important implications for understanding developments in fertility across rich societies more generally.

We combine longitudinal, individual-level register data on the full population of childbearing age for the period 2012–2023 with regional-level information on house prices. This addresses some limitations that were present in previous work on housing and fertility. First, many previous studies were based on aggregate-level data (e.g., Clark, 2012; Dettling & Kearney, 2014; Mulder & Billari, 2010; Simon & Tamura, 2009; Van Wijk, 2024), which does not allow the identification of the micro-level mechanisms through which house prices are linked to fertility. Second, previous work at the individual level has largely been based on data from (panel) surveys (e.g., Atalay et al., 2021; Clark & Ferrer, 2019; Tocchioni et al., 2021), which tends to be hampered by small sample sizes, selective non-response, and panel attrition. Third, where many previous studies have focused on the impact of between-region variation in house prices (e.g., Clark, 2012; Florida et al., 2021; Öst, 2012), we focus in our study on within-region changes in house prices over time. This approach has the advantage that the house price effects that we estimate are not affected by unobserved, time-constant differences between regions. Moreover, focusing on change over time aligns well with the aim of providing insight into the link between the macro-level increase in house prices and decrease in fertility over the past decade. Fourth, in addition to estimating models for the full population, we also estimate parity-specific models and models that only include co-residing couples. These models provide additional insight into how the association between housing market developments and fertility differs by birth order, as well as to what extent effects are driven by the selection into co-residence. Overall, this provides novel insights into the pathways through which rising house prices affect fertility and amplify reproductive inequalities between housing market insiders and outsiders.

## Theory and Hypotheses

The starting point of our theoretical framework is the expectation that increasing house prices will result in a reduction of fertility. This expectation is in line with recent macro-level trends of declining fertility and rising house prices and has frequently been put forward in the demographic literature (Brauner-Otto, 2023; Clark, 2012; Florida et al., 2021). At least three mechanisms may cause a negative house price effect on fertility (see also Flynn, 2017; Van Wijk, 2024). First, rising house prices reduce the accessibility of family-friendly housing to young adults. We use the term “family-friendly housing” as a shorthand for housing with characteristics that are often perceived as most appropriate for raising children, such as large, single-family homes with gardens that are located in child-friendly neighborhoods (Lauster, 2010). As in many other countries, in the Netherlands such family-friendly housing characteristics are more common in owner-occupied homes than in the rental sector (Kullberg, 2016; Statistics Netherlands, 2025). Therefore, rising house prices may make it increasingly difficult for young adults to obtain family-friendly housing. As obtaining family-friendly housing constitutes a prerequisite to having children for many (Chudnovskaya, 2019; Feijten & Mulder, 2002; Kulu & Steele, 2013; Ström, 2010), young adults whose access to family-friendly housing is blocked may decide to postpone childbearing or even give up on having children altogether. Second, rising house prices tend to increase the cost of housing, either in terms of rents or mortgage payments, which leaves potential parents with less financial space to invest in children. Moreover, as housing costs account for a large share of childrearing expenses (Lino et al., 2017), the cost of children is likely to be higher in more expensive housing markets. In line with economic theory (Becker, 1960; Easterlin, 1975), it can be expected that less financial resources to invest in children and a higher cost of children will lower fertility. Third, rising house prices may have an indirect effect on fertility by posing obstacles to other life course transitions (Flynn, 2017). In particular, increases in house prices likely pose obstacles for young adults who wish to leave the parental home (Hochstenbach et al., 2025). In addition, increasing house prices may hamper the formation of (cohabiting or marital) unions, as rising prices may cause a lack of affordable housing that is (perceived to be) appropriate for co-residence with a partner (e.g., housing that has a separate bedroom; housing that is not shared with others).^2^ The delays in leaving the parental home and entering a (co-residing) union with a partner may in turn result in delays in the transition to parenthood and possibly in reductions of completed fertility.

These mechanisms may be particularly relevant in the Netherlands in recent years, as the strong increase in house prices and the overall scarcity of housing has greatly limited the accessibility and affordability of family-friendly housing, particularly for younger cohorts (ABF Research, 2022; Boelhouwer, 2020; Hochstenbach, 2022). This inspires the hypothesis that (hypothesis 1) individuals who live in regions where house prices increased more are less likely to conceive a child.

Whereas the discussion has so far focused on mechanisms that predict a negative relationship between house prices and fertility, an alternative prediction that has gained prominence in the economic literature is that rising house prices will actually increase fertility through increases in home equity (Atalay et al., 2021; Clark & Ferrer, 2019; Dettling & Kearney, 2014; Lovenheim & Mumford, 2013). More specifically, it has been argued that rising house prices increase the level of wealth of existing homeowners. This may in turn improve homeowners’ perceived affluence and possibly increase opportunities to liquefy housing assets through mortgage equity withdrawal, which could facilitate them in fulfilling their fertility desires by increasing the financial resources they have at their disposal.

Previous empirical work has examined the relationship between house prices and fertility both by investigating variation between areas and changes within areas over time. Studies that focused on between-area variation have generally found that fertility is lower in areas where house prices or housing costs are higher. Such negative associations have been reported for municipalities and regions in Sweden (Florida et al., 2021; Öst, 2012, for the 1974 cohort but not the 1956 and 1964 cohorts), for metropolitan areas in the United States (Clark, 2012, for first births but not for completed fertility; Simon & Tamura, 2009), and in a cross-national study in France, Germany, and Italy (Flynn, 2017). However, no between-region relationship between house prices and fertility was found in Austria (Flynn, 2017) and the Netherlands (Van Wijk, 2024).

A problem with these between-area associations is that they may be influenced by unobserved time-constant differences between regions, for example in religiosity or fertility norms. In response to this issue, other studies have focused on examining the relationship between changes in house prices and changes in fertility over time. The findings of these studies have been relatively mixed. Using data on metropolitan areas in the USA, Dettling and Kearney (2014) found that increases in house prices were associated with increases in fertility. In contrast, a recent study in the Netherlands that used regional-level data has shown that fertility declined (more) in regions where house prices increased (more), at least in the post-2010 period (Van Wijk, 2024).

### Homeownership as Mediator

The transition to homeownership is a crucial event in the housing career, and the distinction between renters and homeowners marks a key division on the housing market as well as in the level of wealth accumulation (Boelhouwer, 2020; Hochstenbach, 2022). Living in an owner-occupied home generally comes with housing characteristics that are considered more suitable for raising children, such as large, single-family homes of better quality in child-friendly locations (Ström, 2010). Moreover, homeownership likely signals adult status and may therefore be perceived as a prerequisite for having children in and of itself (Brauner-Otto, 2023). As a result, homeownership can be expected to be positively associated with fertility.

However, the strong surge in house prices over the past decades has made homeownership become increasingly inaccessible to younger cohorts, as illustrated by the widespread use of the term “Generation Rent” to describe today’s young adults (e.g., Timperley, 2020). We expect that young adults’ transition to homeownership is particularly impeded in regions with strongly increasing house prices, forcing them to rely on the rental market instead. Combined with the predicted positive effect of homeownership, this results in the hypothesis that (hypothesis 2) individuals who live in regions where house prices increased more are less likely to conceive a child because they are less likely to be homeowners.

Contrary to this expectation, some older studies have theorized and sometimes found support for a negative relationship between homeownership and fertility, which was interpreted as evidence showing that the cost of homeownership competes with the cost of having children (Courgeau & Lelièvre, 1992). However, we expect that in recent years in rich societies homeownership will be positively related to fertility, in line with recent findings (Chudnovskaya, 2019; Flynn, 2017; Japaridze & Sayour, 2024; Lim, 2021; Öst, 2012; Ström, 2010; Tocchioni et al., 2021) and more general trends toward increases in the economic standards that young adults wish to fulfill before becoming parents (Van Wijk & Billari, 2024).

Moreover, it should be noted that the relationship between homeownership and fertility may run in two directions: homeownership may stimulate fertility, but fertility intentions may also accelerate the transition to homeownership (Kulu & Steele, 2013; Mulder, 2006). Some authors have argued that the lack of affordable housing that characterizes recent times has made the causal pathway running from homeownership to fertility more pertinent than the reverse relationship (Ström, 2010). However, the two directions of causality are difficult to disentangle empirically (Vidal et al., 2017), and in the present paper, we do not aim to make causal claims regarding the relationship between homeownership and fertility.

### Homeownership as Moderator

Rising house prices may affect fertility via other pathways than through blocking the transition to homeownership. The nature of these pathways likely depends on whether individuals are renters or homeowners. For renters, living in an expensive housing market region likely comes with higher housing costs, a lower accessibility of family-friendly housing (e.g., small apartments instead of larger detached or terraced houses), and a lower (perceived) likelihood of entering into homeownership in the near future. In line with the mechanisms stressing the importance of the accessibility and cost of family-friendly housing to fertility decisions, increasing house prices should decrease the fertility of renters.

For homeowners, rising house prices may also come with higher housing costs and a lower accessibility of family-friendly housing, particularly for homeowners in smaller or lower-quality homes who wish to move to another owner-occupied home before having (more) children (Liu et al., 2023). At the same time, however, homeowners may profit from rising house prices as they imply increases in housing wealth. Consistent with the home equity effect, increases in housing wealth may increase homeowners’ fertility, and these effects have been shown to be quite sizeable in previous studies (Atalay et al., 2021; Dettling & Kearney, 2014; Lovenheim & Mumford, 2013). This inspires the hypothesis that (hypothesis 3) increasing house prices decrease the fertility of renters, but have no or a positive effect on the fertility of homeowners.

The moderating role of homeownership in the relationship between rising house prices and fertility is supported by a small literature in economics, which has reported higher fertility among homeowners whose home value had increased in Australia (Atalay et al., 2021), Canada (Clark & Ferrer, 2019), and the USA (Lovenheim & Mumford, 2013). In contrast, increases in home values were associated with lower fertility in China (Liu et al., 2023). However, these studies seem to have been primarily concerned with the estimation of a causal wealth effect on the fertility of homeowners rather than with the impact of rising house prices on fertility patterns in the general population. This focus has, for example, inspired the estimation of separate models for homeowners and renters, which precludes house prices to affect fertility by influencing young adults’ opportunities to transition into homeownership. In addition, choices made in the sample selection in these studies—such as the selection of married couples or of respondents who did not move to another house in the past few years—have further limited the pathways through which house prices may impact fertility. These selection criteria have limited the number of observations for renters in the samples used in previous studies, which has likely contributed to the absence of statistically significant effects of house prices on renters’ fertility in most previous work (Atalay et al., 2021; Clark & Ferrer, 2019; Lovenheim & Mumford, 2013; but see Japaridze & Sayour, 2024).

Finally, in the current context of rapidly increasing house prices, the timing of entry into homeownership—before or after the large house price increases—may matter. Homeowners who bought their home a long time ago, before house prices increased, are more likely to have profited from increases in housing wealth, whereas they are less likely to face high housing costs in terms of mortgage payments. Therefore, increasing house prices can be expected to increase the fertility of longer-term homeowners. In contrast, homeowners who only recently entered into homeownership in more expensive housing markets will have profited from increases in housing wealth to a lesser extent, whereas they are likely tied to high mortgage payments that are needed to finance their recent home purchase in an expensive housing market. Therefore, we expect that (hypothesis 4) the effect of increasing house prices on fertility is more strongly positive for long-term homeowners than for recent homeowners. We are not aware of any previous studies that have tested the moderating role of the duration of homeownership.

### Parity-Specific Effects

So far, it has been assumed that changes in house prices have similar effects on the transition to parenthood and on higher-order births. However, housing conditions may be more strongly related to first than to later births. First births tend to be more easily postponed than later births as biological and social age limits play less of a role (Wood & Neels, 2017). In contrast, considerations regarding fecundity may play a larger role in higher-order births, and births may be spaced together closely by parents who wish to have children who do not differ in age too much. Moreover, parents who already had children in suboptimal housing conditions may form a selective group for whom housing constraints will pose less of a barrier also in the decision to have additional children. Our final hypothesis thus states that (hypothesis 5) the effects of increasing house prices on fertility and the mediating and moderating roles of homeownership will be more pronounced for first than for higher-order births.

## Housing Market and Fertility Developments in the Netherlands

The Dutch housing market has been characterized as a career homeownership regime (Mulder & Billari, 2010). Most people start their housing career in rental housing after they leave the parental home, and the transition into homeownership is made later in the life course by those whose incomes are sufficiently high and stable. Out of the total housing stock in 2022, 57% of housing was owner-occupied (Statistics Netherlands, 2024a). In addition, although declining in recent years the Netherlands still has a relatively large social housing sector (Van Gent & Hochstenbach, 2020): 29% of the housing stock was owned by housing corporations in 2022, whose main purpose is to supply mostly low-income groups with affordable housing. The remaining 14% of homes in 2022 were in the private rental market (Statistics Netherlands, 2024a).

Generous taxation and borrowing arrangements favor households in owner-occupied homes, and this has led to the mortgage debt burden of households in the Netherlands being among the highest of the developed world (International Monetary Fund, 2023). The practice of mortgage equity withdrawal is relatively uncommon in the Netherlands compared to countries like Australia or the UK and has decreased in popularity since the late 1990s in response to changes in mortgage interest deduction tax policies that deterred homeowners from extracting equity from their homes (Haffner et al., 2015). The relative absence of mortgage equity withdrawal may make large positive effects of house prices on the fertility of homeowners less likely in this study compared to most previous studies, as such effects may rely on homeowners’ ability to liquefy increases in housing wealth to facilitate spending on children (Liu et al., 2023). At the same time, increased home equity could still have a positive effect on the fertility of homeowners through increases in perceived wealth, decreases in housing costs resulting from lower loan-to-value ratios, and enhanced opportunities to move to a new home.

Over the past decade, housing in the Netherlands—as in many other rich societies (Galster & Lee, 2021; OECD, 2022)—has become increasingly scarce and expensive, prompting many observers to speak of a “housing crisis” (Hochstenbach, 2022). The housing crisis has particularly hit the current cohort of young adults, who face a choice between taking on a very high mortgage to buy a home (if their employment and financial situation allows) or—given long waiting lists in the social housing sector—to spend a large share of their income on rent in the private rental sector (Hochstenbach, 2022). In 2021, 39% of young adults aged 25 to 34 who were looking for a new home named the price of housing as the most important barrier, which was more than in any other age group (ABF Research, 2022). The lack of affordable housing is not equally experienced across the country, however. Instead, house prices have increased at a much faster rate in the urban areas in the West of the Netherlands than in the less-populated regions closer toward the borders with Belgium and Germany (Van Wijk, 2024).

During the same period that house prices saw their sharp increase, fertility in the Netherlands has declined, from a TFR of 1.80 in 2010 to 1.43 in 2023 (Statistics Netherlands, 2024b). The fertility decline was particularly pronounced among women who are younger than 30 and women who did not yet have children (Van Duin & Feijten, 2023), and mothers’ age at first birth increased from 29.4 in 2010 to 30.3 in 2023 (Statistics Netherlands, 2024b). The present study will contribute to a better understanding of the different ways in which the housing crisis has contributed to this fertility decline.

## Data and Variables

We use data from Statistics Netherlands’ System of Social statistical Datasets (SSD), a system of interlinked registers that contains data on the entire population of the Netherlands (Bakker et al., 2014). From the registers, we derive yearly information on all women aged 16–45 and men aged 16–50 for every year between 2012 and 2023. Persons who live in institutional households (e.g., prisons; nursing homes) are excluded from the analyses.

The dependent variable measures whether an individual had a child in year t + 1.^3^ Thus, whereas the housing variables and the other independent variables are measured in the period 2012–2022, births are measured in the years 2013–2023. Persons who emigrate or pass away during the year in which fertility is measured are excluded from the analyses.

We link these individual-level data to regional data for the 40 NUTS-3 regions of the Netherlands. NUTS-3 regions are constructed from functional relationships between areas and usually comprise a central city and its surrounding service area, with slightly over 400,000 inhabitants on average. Nearly three quarters of all housing moves take place within the same NUTS-3 region (Lennartz et al., 2023), and as such these regions can be argued to provide good approximations of individuals’ housing search area. Moreover, the boundaries of NUTS-3 regions changed only marginally during the study period, providing an appropriate spatial unit for the purpose of our study.

The main regional characteristic of interest is the average sales price of existing dwellings (derived from Statistics Netherlands, 2024c; this includes prices of both single-family and multi-family housing). House prices are measured in the first quarter of year t to approximate the time of conception. House prices are adjusted for inflation to 2022 prices and measured in units of 100,000 euros. We control for a region’s unemployment rate. Moreover, all models control for region fixed effects. Region fixed effects absorb all differences between regions that are constant over time. This implies that the effects of house prices reported in the paper capture how conception risks are associated with changes in regional house prices over time, rather than with the average house price level in a region. Our data show that although house prices increased in all regions during our study period even after adjusting for inflation, the increase was much larger in some regions than in others. For example, house prices in the Greater Amsterdam region increased from 333 thousand euros in 2012 to 605 thousand euros in 2022, while the same period saw a much smaller increase from 211 to 299 thousand in the Zuid-Limburg region. These geographical differences are exploited in this study to investigate the impact of changes in house prices on fertility.

At the individual level, the main independent variable measures whether a person and/or their partner owns the home in which they are living. We code owners of the home and their partners as homeowners, and all other persons as renters. Throughout the paper, we refer to all persons who do not own their home as renters, even though they do not necessarily pay rent. (This implies that persons who are living with their parents, for example, are also designated as renters.)

Our preferred measure of homeownership differs somewhat from another measure of homeownership that is available in the Dutch population registers, which indicates whether a person lives in an owner-occupied home. We prefer the homeownership variable discussed in the main text, because it does not designate individuals who are living in an owner-occupied home that is owned by someone other than them or their partner as homeowners. However, a sensitivity check that used the alternative homeownership variable based on owner-occupation—combined with a separate category for individuals who live with their parents—showed results that are highly similar to those reported in the paper.

Homeownership is measured on the 1st of January of year t. To examine the influence of the duration of homeownership, we create an alternative version of this variable, which distinguishes between homeowners who first bought a home (1) in the past year; (2) between one and three years ago; and (3) more than three years ago. In both homeownership variables, renters are designated as the reference category.

We expect that it is the duration of homeownership in all homes that matters for wealth accumulation, rather than the time in the current home. Therefore, the homeownership duration variable includes past homeownership both in the current and in previous homes. As house prices increased in all regions during our study period, a longer duration of homeownership indicates more opportunities for wealth accumulation, particularly in regions and times where house prices increased more rapidly.

In addition to the controls for unemployment rates at the regional level, all models further control for calendar year dummies and for individual-level age dummies, parity, income quintiles,^4^ main economic activity, highest level of educational attainment,^5^ and migration background. The categorization and distribution of these variables is shown in Table 1 for women.

**Table 1:** Distribution of women across variables in the models, for all person-years included in the analysis

	Did not conceive child		Conceived child		Total	
	N	%^a^	N	%^a^	N	%^b^
Tenure						
Renter	17,849,497	96.2%	710,128	3.8%	18,559,625	56.7%
Homeowner	13,162,945	92.9%	1,005,767	7.1%	14,168,712	43.3%
Income						
First quintile	7,870,254	97.8%	180,513	2.2%	8,050,767	24.6%
Second quintile	7,839,820	95.7%	354,857	4.3%	8,194,677	25.0%
Third quintile	7,386,923	93.4%	518,100	6.6%	7,905,023	24.2%
Fourth quintile	4,855,203	92.0%	421,380	8.0%	5,276,583	16.1%
Fifth quintile	3,060,242	92.7%	241,045	7.3%	3,301,287	10.1%
Main activity						
Employee	17,494,396	93.3%	1,257,619	6.7%	18,752,015	57.3%
Self-employed	2,090,081	94.6%	118,618	5.4%	2,208,699	6.7%
Not employed	4,991,033	94.8%	276,306	5.2%	5,267,339	16.1%
In education	6,436,932	99.0%	63,352	1.0%	6,500,284	19.9%
Educational attainment						
ISCED 0–1 ((pre-)primary education)	1,324,061	95.9%	57,261	4.1%	1,381,322	4.2%
ISCED 2 (lower secondary education)	4,151,973	96.8%	137,435	3.2%	4,289,408	13.1%
ISCED 3 (higher secondary education)	11,705,105	94.8%	637,626	5.2%	12,342,731	37.7%
ISCED 4–6 (tertiary education, bachelor level)	6,091,348	93.1%	449,679	6.9%	6,541,027	20.0%
ISCED 7–8 (tertiary education, master level)	3,155,241	92.1%	272,359	7.9%	3,427,600	10.5%
Level of education unknown	4,584,714	96.6%	161,535	3.4%	4,746,249	14.5%
Migration background						
Born in NL, parents born in NL	22,436,123	94.8%	1,220,255	5.2%	23,656,378	72.3%
Born in NL, parents born in another European country	828,915	95.6%	37,723	4.4%	866,638	2.6%
Born in NL, parents born outside Europe	3,030,921	94.3%	183,963	5.7%	3,214,884	9.8%
Born in another European country	1,550,850	94.8%	84,789	5.2%	1,635,639	5.0%
Born outside Europe	3,165,633	94.4%	189,165	5.6%	3,354,798	10.3%
Number of children						
0	15,967,326	95.4%	762,744	4.6%	16,730,070	51.1%
1	4,117,044	86.5%	640,703	13.5%	4,757,747	14.5%
2	7,462,336	97.1%	223,723	2.9%	7,686,059	23.5%
3	2,617,107	97.8%	60,126	2.2%	2,677,233	8.2%
4	627,153	97.3%	17,500	2.7%	644,653	2.0%
5 or more	221,476	95.2%	11,099	4.8%	232,575	0.7%
Tenure, with duration of homeownership (subsample)						
Renter	11,217,819	95.9%	481,908	4.1%	11,699,727	54.6%
Homeowner, < 1 year	657,365	86.6%	101,798	13.4%	759,163	3.5%
Homeowner, 1–3 years	1,206,317	85.9%	198,029	14.1%	1,404,346	6.5%
Homeowner, > 3 years	7,187,836	94.8%	393,501	5.2%	7,581,337	35.4%
**Continuous variables**	**Mean**	**SD**	**Min**	**Max**		
House prices (in 100,000 euros)	3.106	0.865	1.476	6.517		
Unemployment rate	5.078	1.836	2.122	9.500		
Age^c^	30.884	8.742	16	45		
Year^c^	2018.010	3.174	2013	2023		

^a^Row percentage. ^b^Column percentage. ^c^Age and year are shown as continuous variables for descriptive purposes, but are included in the models using age and year dummies

## Modeling Strategy

All models are run using discrete-time event history analysis, with a logit link and standard errors clustered at the level of individuals. We show the results for women in the main text because information on births is most complete for women, but results for men are very similar and are reported in Appendix. Model 1 includes the effect of regional-level house prices as well as all control variables. In Model 2, we add a dichotomous variable capturing individual-level homeownership, and we estimate to what extent individual-level homeownership mediates the effect of regional house prices. To address the issue of rescaling in logistic regression, we use the KHB method (Karlson et al., 2012). This method keeps constant the error variance of Models 1 and 2, which allows for an unbiased comparison of the house price coefficient before and after adding our homeownership variable. We add a cross-level interaction effect between homeownership on the one hand and regional house prices on the other in Model 3. In Model 4, the dichotomous homeownership variable is replaced by the variable that distinguishes between homeowners depending on the duration of homeownership. The duration of homeownership is interacted with the regional house price variable to examine to what extent the house price effect depends on the moment at which persons entered into homeownership. As a period of at least three years is required to construct the homeowner duration variable, the model that includes this variable is estimated for a subset of persons who have been in the data for at least three years. Specifically, this selection criterion excludes observations in calendar years 2012–2014, women at ages 16 or 17 years old, and migrants who recently arrived in the Netherlands.^6^

In the first set of models we include all births, but in additional parity-specific analyses we run the four models discussed above separately for childless women, women who have one child, and women who have two or more children. In the latter two analyses, we also include a control variable that measures the time since the previous birth, distinguishing between women who had their previous child (1) in the previous year (ref. cat.); (2) two years ago; (3) three years ago; (4) four years ago; (5) five-to-seven years ago; (6) eight-to-eleven years ago; and (7) twelve or more years ago.

Most of the models include all individuals regardless of their household type and partnership status. This is driven by the expectation that housing market conditions may affect fertility by posing obstacles to leaving the parental home and union formation, in line with the indirect effect mechanism discussed above. However, to examine the sensitivity of our findings to this choice, we conduct a robustness check by rerunning the models on a subset of women who were co-residing with a partner on the 1st of January of year t. These models include all different-sex couples where both partners meet the age criteria (i.e., the woman is between 16 and 45 years old and the man is between 16 and 50 years old). The models additionally control for the male partner’s age (using a linear and quadratic term), income, main activity status, level of educational attainment, and migrant background.

In the main analyses, 4.4 million women are included, who contribute 32.7 million person-years to the models (in Model 4, these numbers are reduced to almost 3.7 million women and 21.4 million person-years). Because of the large number of observations in the data, we focus our interpretation on substantive rather than statistical significance. We illustrate the findings of the logistic regression models by calculating predicted probabilities using Stata’s margins command (Williams, 2012). The effects of house prices are captured by calculating the difference in the predicted probability of conception between a situation where house prices are 50,000 euros below the average value and 50,000 euros above the average value. Finally, we use graphs of predicted probabilities to illustrate the cross-level interaction effects.

## Results

Model 1 in Table 2 shows the results of a model that includes the effects of house prices as well as all control variables. Whereas Table 2 (as well as Tables 3 and 4) reports coefficients on the log odds scale of the models, we have calculated predicted probabilities and mention these in the text below to illustrate the substantive meaning of the associations. We find that an increase in house prices is associated with a decrease in the risk of conception, confirming hypothesis 1. Specifically, a regional house price increase of 100,000 euros is associated with a reduction of the probability of conception by 3.1% (the predicted probability of conception decreases from 0.0533 to 0.0516).

**Table 2:** Logit coefficients and standard errors of event history models for women. Dependent variable: conception of a child

	Model 1		Model 2		Model 3		Model 4	
	b	SE	b	SE	b	SE	b	SE
**Regional characteristics**								
House prices (100,000 euros)	− 0.036	0.004	− 0.026	0.004	− 0.054	0.004	− 0.041	0.006
Unemployment rate (%)	− 0.001	0.003	− 0.008	0.003	0.003	0.003	− 0.002	0.003
**Individual-level characteristics**								
Homeowner			0.666	0.002	0.421	0.006		
Duration of homeownership (ref. = renter)								
Homeowner, < 1 year							0.819	0.016
Homeowner, 1–3 years							0.769	0.011
Homeowner, > 3 years							0.199	0.009
Income (ref. = first quintile)								
Second quintile	− 0.078	0.003	− 0.019	0.003	− 0.020	0.003	− 0.012	0.004
Third quintile	0.105	0.004	0.108	0.004	0.107	0.004	0.114	0.005
Fourth quintile	0.257	0.004	0.190	0.004	0.187	0.004	0.189	0.005
Fifth quintile	0.497	0.004	0.377	0.004	0.368	0.004	0.373	0.005
Main activity (ref. = employee)								
Self-employed	0.113	0.003	0.122	0.003	0.122	0.003	0.118	0.004
Not employed	− 0.033	0.003	0.087	0.003	0.083	0.003	0.053	0.003
In education	− 1.113	0.006	− 1.029	0.006	− 1.031	0.006	− 1.024	0.007
Educational attainment (ref. = ISCED 3 (higher secondary education))								
ISCED 0–1 ((pre-)primary education)	0.036	0.006	0.134	0.006	0.132	0.006	0.067	0.007
ISCED 2 (lower secondary education)	0.007	0.004	0.072	0.004	0.071	0.004	0.047	0.004
ISCED 4–6 (tertiary education, bachelor level)	0.046	0.002	0.031	0.002	0.032	0.002	0.012	0.003
ISCED 7–8 (tertiary education, master level)	0.112	0.003	0.127	0.003	0.126	0.003	0.093	0.003
Level of education unknown	− 0.145	0.003	− 0.170	0.003	− 0.165	0.003	− 0.221	0.005
Migration background (ref. = Born in NL, parents born in NL)								
Born in NL, parents born in another European country	− 0.099	0.006	− 0.058	0.006	− 0.058	0.006	− 0.060	0.007
Born in NL, parents born outside Europe	0.101	0.003	0.211	0.003	0.213	0.003	0.228	0.003
Born in another European country	− 0.130	0.004	− 0.048	0.004	− 0.050	0.004	− 0.082	0.005
Born outside Europe	0.243	0.003	0.370	0.003	0.369	0.003	0.321	0.004
Number of children (ref. = 0)								
1	0.975	0.002	0.814	0.002	0.813	0.002	0.840	0.003
2	− 0.333	0.003	− 0.554	0.003	− 0.554	0.003	− 0.494	0.004
3	− 0.438	0.005	− 0.638	0.005	− 0.638	0.005	− 0.572	0.006
4	− 0.110	0.008	− 0.266	0.008	− 0.266	0.008	− 0.194	0.010
5 or more	0.700	0.012	0.583	0.012	0.583	0.012	0.671	0.014
**Cross-level interactions**								
Homeowner*house prices					0.079	0.002		
Homeowner, < 1 year*house prices							− 0.006	0.005
Homeowner, 1–3 years*house prices							0.017	0.003
Homeowner, > 3 years*house prices							0.101	0.003
Constant	− 2.092	0.020	− 2.450	0.021	− 2.319	0.021	− 2.349	0.029
N	32,728,337		32,728,337		32,728,337		21,444,573	
AIC	11,215,187		11,101,292		11,099,652		7,589,126	

All models control for region fixed effects and age and year dummies.

The dichotomous variable that measures individual-level homeownership is added in Model 2. This model shows that homeowners are 80% more likely to conceive a child than renters (the predicted probability is 0.0390 for renters and 0.0699 for homeowners), exposing a strong positive link between homeownership and fertility. Moreover, additional analyses show that in our population of women of childbearing age, women are less likely to be homeowners in regions where house prices have increased more (results not shown). Comparing Model 1 and Model 2 shows that the negative effect of increasing house prices attenuates after adding individual-level homeownership to the model. Formal mediation analyses based on the KHB method show that women’s lower propensity to be homeowners in more expensive housing market regions can explain 11% of the negative effect of rising house prices. Overall, this supports the hypothesis (hypothesis 2) that the lower propensity to be homeowners explains part of women’s lower fertility in more expensive housing market regions. At the same time, much of the association between rising house prices and lower fertility is not explained by individual-level homeownership, suggesting other factors are needed to explain this association.

**Fig. 1:**
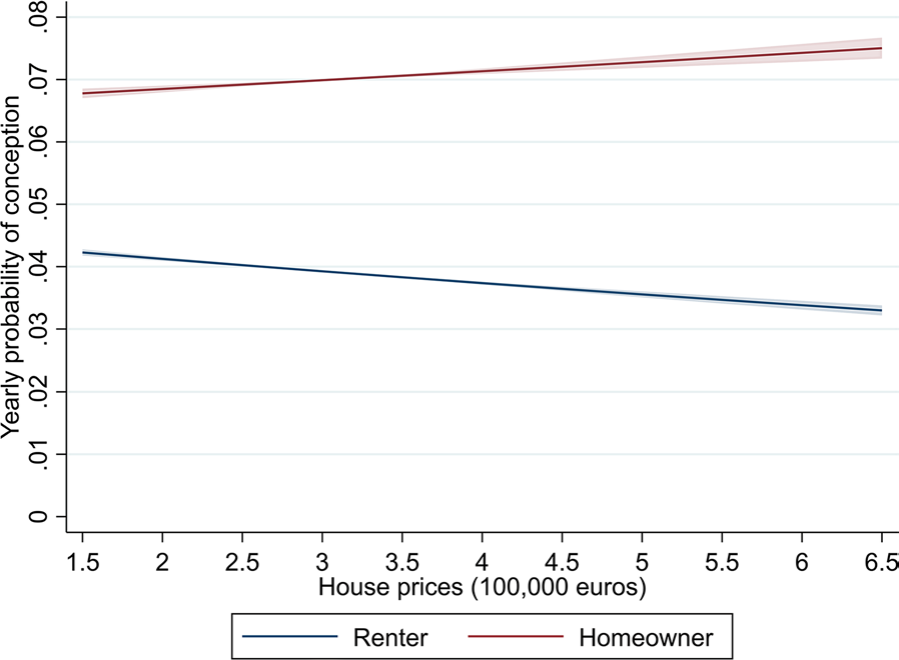
Predicted probability of conception by tenure and regional house prices, for women. Based on Model 3 in Table 2

In Model 3, a cross-level interaction effect is added between individual-level homeownership and regional house prices. The interaction effect is positive and relatively large, showing that the association between regional house prices and fertility clearly differs by individual-level homeownership. The results of Model 3 are illustrated in Fig. 1, which shows that increasing house prices are associated with a higher probability of conception for women who are homeowners, but a lower probability of conception for women who rent their home. The predicted probabilities show that women who live in regions where house prices increased by 100,000 euros more have a 2.1% higher probability of conception when they are homeowners, but a 4.8% lower probability when they are renters. These findings show clear support for hypothesis 3.

**Fig. 2:**
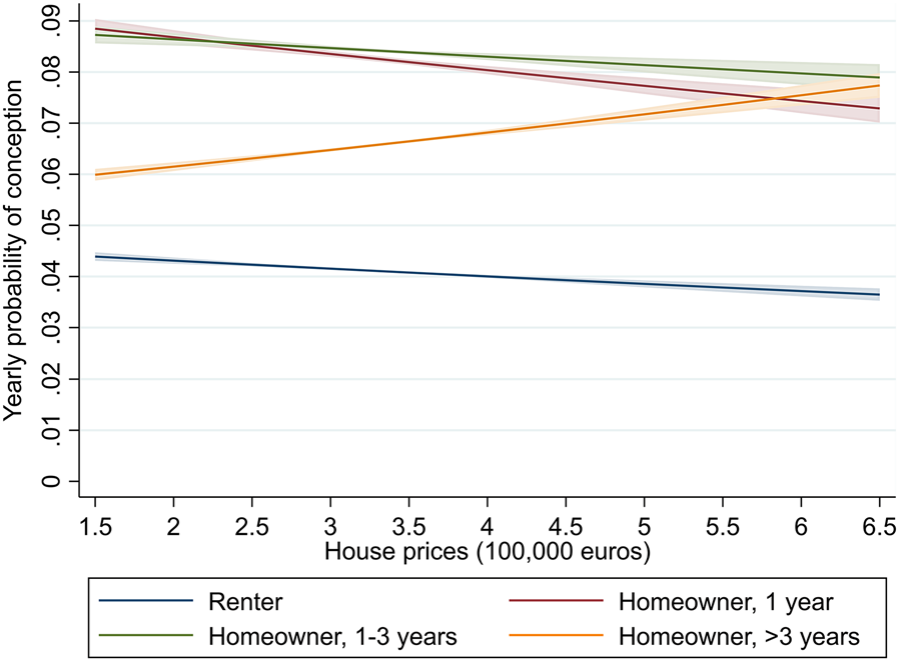
Predicted probability of conception by duration of homeownership and regional house prices, for women. Based on Model 4 in Table 2

Model 4 provides further insight into the role of homeownership as moderator of the house price effects on fertility by distinguishing between groups of homeowners based on the duration of homeownership. Recall that this model is estimated for a smaller population than the previous models, as it only includes persons who have been observed for three or more years. The main effect of homeownership duration in Model 4 indicates that the probability of conception is particularly high among women who transitioned into homeownership in the past three years. Moreover, the interaction effect between the duration of homeownership and regional house prices shows that the effect of regional house prices on fertility depends upon the duration of homeownership. The results are illustrated in Fig. 2. This figure shows that for women who bought their home in the past year, rising house prices are negatively associated with conception risks. Specifically, an increase in house prices of 100,000 euros is associated with a decrease in the probability of conception of 3.8% among women who bought their home in the last year, an effect that is similar in size as that for renters. For women who bought their home between one and three years ago, the effect of rising house prices on fertility is still negative but weaker than for renters and more recent homeowners (a 100,000-euro increase in house prices is associated with a 2.0% decrease in the probability of conception). Finally, women who have been homeowners for more than three years are more likely to conceive a child if they live in regions where house prices increased more: for this group, an increase in house prices of 100,000 euros is associated with an increase in the probability of conceiving a child of 5.3%. Overall, these results provide strong support for hypothesis 4, showing that the effect of rising house prices on fertility depends on the duration of homeownership.^7^

### Differences by Parity

In Table 3, the results of Models 1–4 are shown separately for first, second, and third and higher-order births. Model 1 shows that increasing house prices are associated with decreases in the probability of conceiving a first and a third or later birth, in line with the models on the full population. In contrast, house prices have little-to-no effect on second birth rates.

The effect of homeownership in Model 2 also differs by parity. Homeownership is strongly positively associated with first births, but fertility differences between homeowners and renters are much smaller for second births, and renters are more likely to conceive a third or later child than homeowners. Homeownership is found to strongly mediate the effect of house prices on fertility for first births: the lower propensity of childless women to be homeowners in housing market regions where the prices have increased more can fully explain the negative effect of rising house prices. In contrast, the evidence for mediation by homeownership is largely absent for higher-order births.^8^

Interaction effects between homeownership and house prices are added to the parity-specific models in Model 3. The interaction effect between changes in house prices and homeownership is found only for first births, where it is relatively large. The results indicate that childless women who own their homes are more likely to become mothers in regions where house prices increased more, but childless women who rent their home are less likely to become mothers if house prices increased more. In contrast, the effect of house price increases on higher-order births does not seem to differ substantially between homeowners and renters.

Model 4 again explores the role of the duration of homeownership. The models indicate that the effects of increasing house prices are more strongly positive (or less strongly negative) for long-term homeowners than for recent homeowners at all parities, but again the evidence is most convincing for first births.

To conclude, the parity-specific models show that the effects of increasing house prices on fertility and the mediating and moderating role of homeownership are generally more pronounced for first than for higher-order births, in line with hypothesis 5. For first births, increasing house prices appear to constitute a barrier to childbearing as they block the transition to homeownership and decrease the fertility of renters, whereas rising house prices increase the first birth rate among long-term homeowners. The house price effects are generally weaker and less consistent for higher-order births, although some evidence is found for a negative effect of increasing house prices on third and later births as well as a relative decrease in higher-order births among recent homeowners in expensive housing markets compared to longer-term homeowners.

**Table 3:** Logit coefficients and standard errors of event history models for women, by parity. Dependent variable: conception of a child

	Model 1		Model 2		Model 3		Model 4	
	b	SE	b	SE	b	SE	b	SE
**First births**								
**Regional characteristics**								
House prices (100,000 euros)	− 0.037	0.006	− 0.006	0.006	− 0.034	0.006	− 0.010	0.009
**Individual-level characteristics**								
Homeowner			1.084	0.003	0.825	0.009		
Duration of homeownership (ref. = renter)								
Homeowner, < 1 year							1.199	0.019
Homeowner, 1–3 years							1.171	0.016
Homeowner, > 3 years							0.525	0.015
**Cross-level interactions**								
Homeowner*house prices					0.083	0.003		
Homeowner, < 1 year*house prices							− 0.006	0.006
Homeowner, 1–3 years*house prices							0.014	0.005
Homeowner, > 3 years*house prices							0.122	0.004
N	16,730,070		16,730,070		16,730,070		10,402,313	
AIC	5,362,250		5,210,956		5,210,099		3,522,281	
**Second births**								
**Regional characteristics**								
House prices (100,000 euros)	− 0.012	0.007	− 0.010	0.007	− 0.007	0.007	− 0.022	0.011
**Individual-level characteristics**								
Homeowner			0.290	0.004	0.305	0.012		
Duration of homeownership (ref. = renter)								
Homeowner, < 1 year							0.451	0.030
Homeowner, 1–3 years							0.412	0.021
Homeowner, > 3 years							0.215	0.016
**Cross-level interactions**								
Homeowner*house prices					− 0.005	0.004		
Homeowner, < 1 year*house prices							− 0.027	0.009
Homeowner, 1–3 years*house prices							− 0.019	0.006
Homeowner, > 3 years*house prices							0.010	0.005
N	4,757,747		4,757,747		4,757,747		3,251,822	
AIC	2,973,174		2,966,348		2,966,348		2,020,245	
**Third and later births**								
**Regional characteristics**								
House prices (100,000 euros)	− 0.045	0.009	− 0.045	0.009	− 0.038	0.010	− 0.048	0.014
**Individual-level characteristics**								
Homeowner			− 0.306	0.005	− 0.241	0.015		
Duration of homeownership (ref. = renter)								
Homeowner, < 1 year							0.228	0.051
Homeowner, 1–3 years							0.063	0.034
Homeowner, > 3 years							− 0.297	0.019
**Cross-level interactions**								
Homeowner*house prices					− 0.021	0.005		
Homeowner, < 1 year*house prices							− 0.065	0.015
Homeowner, 1–3 years*house prices							− 0.052	0.010
Homeowner, > 3 years*house prices							− 0.025	0.006
N	11,240,520		11,240,520		11,240,520		7,790,438	
AIC	2,383,895		2,379,666		2,379,647		1,664,122	

All models control for region fixed effects, the regional unemployment rate, income, main economic activity, educational attainment, migration background, and age and year dummies. The models that predict second births furthermore control for time since first birth, and the models that predict third and later births control for time since previous birth and number of children.

### Couple-Level Models

In a final step, we rerun Models 1–4 for the population of women who are in a co-residing partnership with a male partner. These models additionally control for the socioeconomic and demographic characteristics of the partner. This additional analysis allows us to examine whether housing developments affect fertility indirectly through union formation and stability, or whether the housing market situation also has a direct effect on the fertility outcomes of couples. The results of this exercise are shown in Table 4. In general, the direction of the house price effect and the mediating and moderating roles of homeownership are similar to the results reported above, but the magnitude of the effects decreases when focusing on co-residing couples. First, rising house prices are associated with a lower risk of conception also when selecting co-residing couples, although the effect is somewhat smaller than in the full-population models. Second, although co-residing couples are more likely to conceive a child when they own their home, fertility differences between renters and homeowners are smaller in the couple-level models than in the full-population models. The lower probability of women to be homeowners in regions with strongly rising house prices can explain only a small (3%) part of the negative effect of rising house prices on fertility when focusing on couples. Third, the interaction effect between homeownership and house prices is weaker in the couple-level models than in the full-population models, although the direction remains the same. There is no evidence that rising house prices increase the fertility of co-residing homeowners in Model 3. However, when distinguishing between different groups of homeowners based on the duration of homeownership in Model 4 we find that the effects of rising house prices differ between recent and longer-term homeowners in similar ways in the couple models as they did in the full-population models. In particular, in regions where house prices increased more, couples who recently became homeowners decrease their fertility, whereas couples who have been homeowners for a longer time are slightly more likely to conceive a child in such regions.

**Table 4:** Logit coefficients and standard errors of event history models for women who co-reside with a male partner. Dependent variable: conception of a child

	Model 1		Model 2		Model 3		Model 4	
	b	SE	b	SE	b	SE	b	SE
**Regional characteristics**								
House prices (100,000 euros)	− 0.029	0.005	− 0.025	0.005	− 0.033	0.005	− 0.026	0.007
**Individual-level characteristics**								
Homeowner			0.191	0.002	0.146	0.008		
Duration of homeownership (ref. = renter)								
Homeowner, < 1 year							0.354	0.017
Homeowner, 1–3 years							0.376	0.013
Homeowner, > 3 years							− 0.012	0.011
**Cross-level interactions**								
Homeowner*house prices					0.014	0.002		
Homeowner, < 1 year*house prices							− 0.047	0.005
Homeowner, 1–3 years*house prices							− 0.028	0.004
Homeowner, > 3 years*house prices							0.039	0.003
N	16,164,526		16,164,526		16,164,526		11,009,729	
AIC	7,462,229		7,456,012		7,455,979		5,099,546	

All models control for region fixed effects, the regional unemployment rate, income, main economic activity, educational attainment, migration background, number of children, age and year dummies, partner’s income, partner’s main economic activity, partner’s educational attainment, partner’s migration background, and partner’s age (linear and quadratic).

## Discussion

House prices have increased rapidly over the past decade in many rich societies, leading to a growing lack of affordable family-friendly housing (Galster & Lee, 2021; OECD, 2022). However, only a small number of studies have examined the consequences of housing affordability problems for fertility, and the individual-level mechanisms that link house prices to fertility have remained largely unknown. The present study has aimed to address this research gap by exploiting the large regional differences in house price developments in the Netherlands, a country that witnessed a dramatic increase in house prices over the past decade, as well as a steady decline in fertility.

Two main consequences of rising house prices for fertility emerged from our study. First, *rising house prices have likely contributed to the postponement of parenthood and the overall fertility decline observed after 2010*. Our findings suggest that increasing house prices reduce fertility by blocking young adults’ access to homeownership, a pathway that has been overlooked in many previous studies that have separately analyzed renters and homeowners. In addition, rising house prices are associated with decreased fertility among renters and recent homeowners. These findings are in line with mechanisms that predict declines in fertility in response to increasing housing costs and an inaccessibility of family-friendly housing. In addition, part of the declining fertility resulting from rising house prices may result from an indirect effect that runs via parental home leaving and union formation processes (Flynn, 2017), as we found that house price effects were generally weaker in models that included only co-residing couples.

The size of the association between rising house prices and declining fertility deserves some further reflection. In the models that estimated an effect of house prices on the fertility of the full population, a 100,000-euro increase in house prices was associated with a drop in the probability of conception of around 3%. Although this effect may seem small at first sight, it should be kept in mind that such house price increases were not uncommon in the past decade—in fact, house prices increased by more than 130,000 euros between 2010 and 2022 in the Netherlands even after adjusting for inflation (own calculations based on Statistics Netherlands, 2024c). This suggests that rising house prices may offer an important explanation for the fertility decline in the Netherlands, in line with previous conclusions based on aggregate-level data by Van Wijk (2024). However, such conclusions need to be interpreted with care, as they require strong assumptions regarding both the causal nature of the within-region house price effect and the translation of individual-level results into macro-level conclusions (Billari, 2015).

The observation that the individual-level relationships found in the current study align well with macro-level fertility developments lends further support to the suspected contribution of house price increases to macro-level fertility decline. At the macro-level, recent evidence has shown that the decline in fertility in the Netherlands since 2010 was most pronounced among younger age groups and for first births (Van Duin & Feijten, 2023), similar to patterns found in other rich societies (e.g., Guzzo & Hayford, 2023; Hellstrand et al., 2020). At the individual level, our results show that negative house price effects were generally more pronounced for first than for higher-order births. A reason could be that first births are more easily postponed and therefore more affected by structural constraints than higher-order births (Wood & Neels, 2017). Alternatively, those who had a first child in suboptimal housing conditions could constitute a selective group for whom housing considerations play less of a role in fertility decisions, which would also result in an effect of housing on first but not on higher-order births. Moreover, it is particularly the recent cohorts of young adults who experienced the detrimental consequences of the housing crisis, resulting in strong declines in homeownership among those in their 20s and early 30s (Hochstenbach, 2022). As our results show that rising house prices are particularly associated with decreasing fertility of renters, this suggests that younger cohorts’ childbearing decisions will be impeded most by housing market constraints. Taken together, this suggests that the inaccessibility of family-friendly, owner-occupied housing and the high costs of housing provide important explanations for the declining first birth rates of recent younger cohorts.

Second, *rising house prices may widen fertility differences between housing market insiders and outsiders*. We found that house prices have different effects among renters and homeowners, in line with the previous work (Atalay et al., 2021; Clark & Ferrer, 2019; Dettling & Kearney, 2014; Lovenheim & Mumford, 2013). For renters, increasing house prices decrease fertility, most likely by blocking entry into homeownership and by increasing housing costs, as discussed above. For homeowners, however, increasing house prices increase fertility, in line with the home equity argument that suggests that existing homeowners benefit from rising house prices through increases in housing wealth. We found that this positive home equity effect is stronger for homeowners who bought a home several years ago, consistent with expectations that longer-term homeowners are the ones who profit most from increasing house prices. In contrast, recent homeowners have lower fertility if they bought a home in a region where house prices increased more, possibly because they are constrained by the high cost of their newly-acquired mortgages—or, alternatively, by the housing characteristics on which they compromised (e.g., dwelling size) in order to be able to afford their new home.

In times of rapidly rising house prices, the pattern that emerges from these findings is one in which fertility outcomes are dependent on whether persons can afford to make the transition into homeownership, as well as whether they were able to profit from increases in housing wealth. Therefore, the housing crisis likely amplifies reproductive inequality between housing market insiders and outsiders. These inequalities exist both between and within birth cohorts. Regarding inequalities between cohorts, today’s younger cohorts have been able to profit to a much more limited extent from the recent house price increases than older cohorts, and are more often stuck in expensive housing in the private rental sector. Our findings suggest that these differences in housing market opportunities translate into different fertility outcomes. Regarding inequalities within cohorts, rising house prices may amplify other socioeconomic inequalities in fertility outcomes, as characteristics such as a person’s income or the financial resources invested by one’s parents will increasingly determine whether people are able to purchase a home that is perceived to be suitable for having children (see also Van Wijk & Billari, 2024).

Our finding of a negative association between house prices and fertility partially deviates from previous studies that focused on the association between within-region changes in house prices and fertility outcomes, some of which have concluded that rising house prices increase fertility (Atalay et al., 2021; Clark & Ferrer, 2019; Dettling & Kearney, 2014; Lovenheim & Mumford, 2013; but see Liu et al., 2023; Van Wijk, 2024). This divergence may first of all result from methodological differences between studies. In particular, the choice of previous studies to analyze renters and homeowners separately and to include only co-residing or married respondents has likely concealed potential pathways through which rising house prices decrease fertility. In addition, our findings may differ from those of previous studies because of differences in study contexts. For example, in contrast to most previous studies’ country contexts, mortgage equity withdrawal has been relatively uncommon in the Netherlands in recent years (Haffner et al., 2015), which likely makes it more difficult for homeowners to liquefy increases in housing wealth and use it for expenditures on children. This difference may explain why the home equity effect in our study is smaller than in the previous work (Liu et al., 2023). Moreover, the strong increases in house prices in the Netherlands over the past decade combined with the stagnation of the incomes of young adults (Hammer et al., 2022) may have produced a context in which the lack of affordable and family-friendly housing constitutes a particularly large barrier to childbearing (Van Wijk, 2024). Possibly, this has made negative effects of house prices on fertility more pronounced in the present study than in previous ones.^9^ However, it should be noted that house prices have risen dramatically over the past decade in many rich societies, which may imply that recent work in other countries will also be more likely to find a link between rising house prices and falling fertility.

Some limitations of this study should be noted, which provide important avenues for future research. First, we are not able to make definitive claims about the direction of causality behind the relationship between house prices and fertility. For example, unobserved time-varying factors such as the prevalence of family-friendly norms may affect both fertility and house prices. Moreover, we cannot rule out the possibility that selective mobility has influenced the results of our study. If individuals who plan to have children in the near future more often move to regions where houses are cheaper, this could (partly) explain the negative house price effects in our study. Such concerns are to some extent mitigated by the use of relatively large regions to measure house prices: as most moves take place within these regions, selective mobility hopefully has only limited impact on our findings (Simon & Tamura, 2009). However, future work should test this assumption by investigating the relationship between house prices, mobility decisions, and fertility more explicitly. Second, future research would benefit from a longer-term perspective on the relationship between house prices and fertility. Doing so will allow a more encompassing incorporation of indirect effects on fertility that run via other life course transitions such as leaving the parental home and union formation and dissolution, which likely take some years to transpire. Such longer-term studies could also help answering the question to what extent current declines in fertility in response to housing market constraints are “merely” postponement effects that will be recuperated at older ages, when housing conditions improve or housing prerequisites are relaxed, or whether housing markets will also affect completed fertility. Third, our focus on relatively large NUTS-3 regions masks variation in house prices and house price developments at a smaller scale, the impact of which could be examined by exploring relationships between housing market characteristics and fertility in smaller geographical areas. Fourth, like much of the previous literature on housing and fertility, the present study has focused on house prices and homeownership. However, many other housing characteristics likely influence fertility decisions, and future studies should incorporate housing aspects such as rent levels, type of rental contracts, energy efficiency, property sizes, and location. Finally, it is up to future research to test whether the current findings also hold beyond the context of the Netherlands. Answering these questions will help housing to take on a more central position in the demographic literature, a much-needed shift at a time when affordable and family-friendly housing is becoming increasingly scarce across rich societies.

## Data Availability

The data that support the findings of this study are available from Statistics Netherlands but restrictions apply to the availability of these data, which are not publicly available.

## Appendix

See Table

**Table 5:** Logit coefficients and standard errors of event history models for men. Dependent variable: conception of a child

	Model 1		Model 2		Model 3		Model 4	
	b	SE	b	SE	b	SE	b	SE
**Regional characteristics**								
House prices (100,000 euros)	− 0.042	0.004	− 0.033	0.004	− 0.056	0.004	− 0.042	0.006
**Individual-level characteristics**								
Homeowner			0.556	0.002	0.369	0.007		
Duration of homeownership (ref. = renter)								
Homeowner, < 1 year							0.777	0.016
Homeowner, 1–3 years							0.734	0.012
Homeowner, > 3 years							0.170	0.009
**Cross-level interactions**								
Homeowner*house prices					0.060	0.002		
Homeowner, < 1 year*house prices							− 0.010	0.005
Homeowner, 1–3 years*house prices							0.001	0.003
Homeowner, > 3 years*house prices							0.068	0.003
N	39,447,679		39,447,679		39,447,679		26,082,276	
AIC	11,272,307		11,202,264		11,201,368		7,666,985	

All models control for region fixed effects, the regional unemployment rate, income, main economic activity, educational attainment, migration background, number of children, and age and year dummies.

5

## Footnotes

1 For this simple count we included Demography; Demographic Research; European Journal of Population; Population and Development Review; Population, Space and Place; and Population Studies.

2 Alternatively, partners in expensive housing markets may decide to move in together to minimize housing costs. We are not aware of any recent studies that distinguish between these two possibilities.

3 Models that used a two-year instead of a one-year time lag between the measurement of the independent variables and fertility outcomes yielded very similar results to those reported here.

4 Income is measured as the annual personal income in year t-1, which ensures that incomes are fully measured before conception. Income quintiles are constructed for the combined population of men and women.

5 A separate category is added for persons whose level of education is unknown in the registers, which happens when the person attended education at an institute that was not included in the registers at that time.

6 In an additional analysis, we estimated Models 1, 2, and 3 for the subset of person-years that are included in Model 4. The results of this analysis are highly similar to those for the full population, which indicates that the selection of person-years in Model 4 does not impact the results.

7 A robustness check that only includes women who are living in the same region as they did three years ago shows similar results to those reported here, which provides some evidence that our findings are not driven by recent selective migration between regions.

8 The interpretation of the mediation models in the parity-specific analyses is complicated by the finding that the relationship between house prices and homeownership differs by parity. Childless women are less likely to own their home in regions where house prices increased more. In contrast, mothers are somewhat more likely to own their home in regions where house prices have increased more.

9 Indeed, OECD (2025) data show that the average annual increase in the house-price-to-income ratio during our study period was about twice as large as the increase during the periods studied by Clark and Ferrer (2019) and Dettling and Kearney (2014). However, the house-price-to-income ratio increase in our study period was similar to that in the period studied by Atalay et al. (2021).
